# Malaria trends in districts that were targeted and not-targeted for seasonal malaria chemoprevention in children under 5 years of age in Guinea, 2014–2021

**DOI:** 10.1136/bmjgh-2023-013898

**Published:** 2024-02-26

**Authors:** Donal Bisanzio, Mamadou Sitan Keita, Alioune Camara, Timothée Guilavogui, Thierno Diallo, Hamidou Barry, Adam Preston, Lamine Bangoura, Eliane Mbounga, Lia S Florey, Jean-Luc Taton, Aissata Fofana, Richard Reithinger

**Affiliations:** 1 RTI International, Washington, District of Columbia, USA; 2 RTI International, Conakry, Guinea; 3 Programme National de la Lutte contre le Paludisme, Ministère de la Santé et de l'Hygiène Publique, Conakry, Guinea; 4 Ministère de la Santé et de l'Hygiène Publique, Conakry, Guinea; 5 RTI International, Fort Collins, Colorado, USA; 6 President's Malaria Initiative, US Agency for International Development, Conakry, Guinea; 7 US Agency for International Development, Washington, District of Columbia, USA

**Keywords:** Malaria, Prevention strategies, Control strategies, Public Health

## Abstract

**Background:**

Seasonal malaria chemoprevention (SMC) is a main intervention to prevent and reduce childhood malaria. Since 2015, Guinea has implemented SMC targeting children aged 3–59 months (CU5) in districts with high and seasonal malaria transmission.

**Objective:**

We assessed the programmatic impact of SMC in Guinea’s context of scaled up malaria intervention programming by comparing malaria-related outcomes in 14 districts that had or had not been targeted for SMC.

**Methods:**

Using routine health management information system data, we compared the district-level monthly test positivity rate (TPR) and monthly uncomplicated and severe malaria incidence for the whole population and disaggregated age groups (<5 years and ≥5 years of age). Changes in malaria indicators through time were analysed by calculating the district-level compound annual growth rate (CAGR) from 2014 to 2021; we used statistical analyses to describe trends in tested clinical cases, TPR, uncomplicated malaria incidence and severe malaria incidence.

**Results:**

The CAGR of TPR of all age groups was statistically lower in SMC (median=−7.8%) compared with non-SMC (median=−3.0%) districts. Similarly, the CAGR in uncomplicated malaria incidence was significantly lower in SMC (median=1.8%) compared with non-SMC (median=11.5%) districts. For both TPR and uncomplicated malaria incidence, the observed difference was also significant when age disaggregated. The CAGR of severe malaria incidence showed that all age groups experienced a decline in severe malaria in both SMC and non-SMC districts. However, this decline was significantly higher in SMC (median=−22.3%) than in non-SMC (median=−5.1%) districts for the entire population, as well as both CU5 and people over 5 years of age.

**Conclusion:**

Even in an operational programming context, adding SMC to the malaria intervention package yields a positive epidemiological impact and results in a greater reduction in TPR, as well as the incidence of uncomplicated and severe malaria in CU5.

**WHAT IS ALREADY KNOWN ON THIS TOPIC**

- When effectively deployed, seasonal malaria chemoprevention (SMC) has shown to reduce childhood malaria by up to 75% in areas of high, seasonal malaria transmission.

**WHAT THIS STUDY ADDS**

- Using routinely collected data from Guinea’s health management information system, we compared the compound annual growth rate (CAGR) of malaria-related outcomes in 14 districts that had or had not been targeted for SMC between 2015 and 2021.
- Compared with non-SMC districts, a statistically lower CAGR in all-age number of clinical malaria cases and clinical malaria test positivity rate (TPR), as well as the incidence of uncomplicated and severe malaria incidence was observed in SMC districts. But for the number of clinical malaria cases, this difference in CAGR between SMC and non-SMC district was observed for both children under 5 years of age and people above 5 years of age.

**HOW THIS STUDY MIGHT AFFECT RESEARCH, PRACTICE OR POLICY**

- Our analyses also show that routine health management information system data and the CAGR approach can be used to continuously monitor malaria intervention effectiveness against standard malariometric indicators.
- Our results provide evidence to support that—even in an operational programming context—adding SMC to the comprehensive package of malaria interventions yields a positive epidemiological impact and results in a greater reduction in TPR, as well as the incidence of uncomplicated and severe malaria in CU5.

## Introduction

Malaria is the leading cause of morbidity and mortality in Guinea, with 2 422 445 confirmed cases and 1029 deaths reported in 2021.^1^ Over the past decade, Guinea’s National Malaria Control Programme (NMCP)—in collaboration with bilateral, multilateral and non-governmental partners—has scaled up malaria prevention and control efforts, including rapid diagnostic tests (RDTs), artemisinin-based combination therapies, intermittent preventive treatment for pregnant women with sulfadoxine–pyrimethamine (SP) and insecticide-treated nets.^2^ Additionally, in 2015, the NMCP piloted and then rolled out seasonal malaria chemoprevention (SMC) in children aged 3–59 months (CU5). Recommended by the WHO since 2012 for countries with seasonal malaria transmission,^3^ SMC is the monthly administration of a single dose of SP and three daily doses of amodiaquine (AQ) (SP-AQ) to CU5 during the peak malaria transmission season. SMC can reduce the incidence of clinical malaria in the 28 days following administration by up to 75% when effectively deployed^4–7^; depending on the length of the rainy season, 3–5 cycles of SMC are conducted.^8^ As of 2021, 13 countries had adopted SMC (ie, Benin, Burkina Faso, Cameroon, Chad, Gambia, Ghana, Guinea, Guinea Bissau, Mali, Niger, Nigeria, Senegal and Togo) at different scales of implementation.^8^

Clinical trials and meta-analyses have demonstrated the efficacy of SMC to reduce malaria incidence during the intervention period and parasitaemia prevalence at the end of the transmission season, and suggest a positive impact on all-cause mortality.^4–7^ There is also some evidence that SMC positively affects other health outcomes (eg, anaemia and malnutrition), but such effects have not always been significant across studies.^7 9–14^ Monthly administration of SP-AQ was found to be the drug regimen with the highest efficacy,^15–17^ and using community health workers (CHWs) to deliver SMC was shown to be more cost-effective than using facility-based nurses, immunisation outreach clinics or outreach trekking teams.^18 19^

While there is strong evidence of the effect of SMC in rigorously conducted academic research studies, evidence of the protective effect of SMC in programmatic contexts is more limited.^7 20 21^ This type of evaluation is challenging but very necessary—indeed, the effectiveness of interventions in the context of public health programmes often differs from the efficacy measured in academic research studies, because of operational challenges when interventions are implemented at scale.^22 23^

The aim of the analyses presented here was to assess the programmatic impact of SMC in Guinea’s context of scaled up malaria intervention programming, specifically by comparing malaria-related outcomes in districts that had or had not been targeted for SMC between 2015 and 2021. Additionally, to do so, we wanted to use a methodological (ie, using data routinely collected by Guinea’s health management information system (HMIS)) and an analytical approach (ie, using the compound annual growth rate, CAGR) that is simple and would allow the NMCP to readily monitor SMC effectiveness against standard malariometric indicators as it continues to expand the intervention across the country.

## Methods

### Study setting

The Republic of Guinea is located in West Africa: it covers a total land area of 245 860 km^2^ and has an estimated population of 13.5 million people. The country is divided into 8 major administrative regions, which are further divided into 38 préfectures (districts), 5 of which comprise the urban areas around the capital city, Conakry. Districts are the main administrative unit where many of the public services are planned, managed and implemented, including for health and malaria. Guinea’s climate is tropical and humid with a wet (June–November) and a dry season (December–May); the rainy season is followed by a peak malaria transmission season, with the highest malaria case count typically observed between July and November/December. Approximately 95% of malaria cases in Guinea are caused by *Plasmodium falciparum*, the principal vectors being *Anopheles gambiae*, *Anopheles funestus* and *Anopheles arabiensis*.^1 24 25^

### SMC in Guinea

Guinea introduced SMC initially in 6 districts in 2015, scaling up to 17 districts by 2021, with 4 monthly community-based SMC campaigns (cycles) conducted during the peak malaria transmission season (generally from July to October). The NMCP implements SMC in partnership with nongovernmental and international organisations that support the successful execution of Guinea’s National Malaria Strategic Plan and its activities. As per WHO guidance,^8^ the recommended drug regimen for SMC is SP-AQ. CHWs (existing ones as well as ones mobilised specifically for the SMC campaign) administer SP and the first dose of AQ to eligible children and give the remaining two daily doses of AQ to the caregiver.

Cycles of SMC are conducted each year—once every 4 weeks during peak malaria transmission season. SMC usually starts in late July, but the exact starting date fluctuates every year depending on several factors, including logistical considerations and rainfall. The target population for SMC comprises all CU5, excluding those with known allergies to SP or AQ, those under cotrimoxazole treatment, and those severely ill or experiencing a presumptive malaria episode. Children 3–11 months of age receive one 25 mg dose of sulfadoxine, one 12.5 mg dose of pyrimethamine and three doses of 75 mg AQ given over the course of three consecutive days; children 12–59 months of age receive double doses of SP and AQ. The children’s vaccination booklets are consulted to help determine their age.

In teams of two, CHWs go door-to-door to every household; sometimes they organise distribution sessions at gathering venues such as markets, churches, dwellings, mosques and fields. After explaining the SMC strategy (objective, rationale, risks and benefits, treatment instructions) to the caregiver, they administer treatments, with any children with malaria or danger signs referred to the closest health centre. CHWs fill in forms where they indicate the number of treatments administered in every household, and they keep updated stock management sheets. Drugs are supplied through the Ministry of Health (MOH) to the health centres, who then allocate them to the CHWs. Nurses in health centres are responsible for coordinating CHWs’ work and for collecting SMC forms. Supervisory visits are conducted by nurses and district health authorities.

### Malaria data

In the aftermath of the 2014–2016 Ebola outbreak, the MOH established a strategic plan to strengthen its routine surveillance system, which led to the adoption of the District Health Information Software (DHIS) 2, an open source, online software application as the national HMIS platform for monthly aggregate data from health facilities. DHIS2 is managed by Guinea’s Système National d’Information Sanitaire (SNIS) and has been rolled out nationally^26^; it is complemented by an infectious disease response and surveillance platform that reports on aggregate disease surveillance and individual case surveillance of epidemic-prone diseases. The NMCP uses DHIS2 to store the monthly aggregate epidemiological surveillance data collected, including for all outpatients and inpatients seen, suspected clinical malaria cases seen, suspected clinical malaria cases tested, malaria cases confirmed and malaria cases treated.^26^ Monthly numbers of tested clinical malaria cases, confirmed positive clinical cases and severe malaria cases are disaggregated by age group (<5 years of age (<5 years), ≥5 years of ages (≥5 years)). Data for CU5 and people ≥5 years of age (PO5) were downloaded for January 2014 to November 2021 from the SNIS portal; we also downloaded the estimated catchment population of each health facility for each year.

### Study area, study population and data analysis

**Figure 1:**
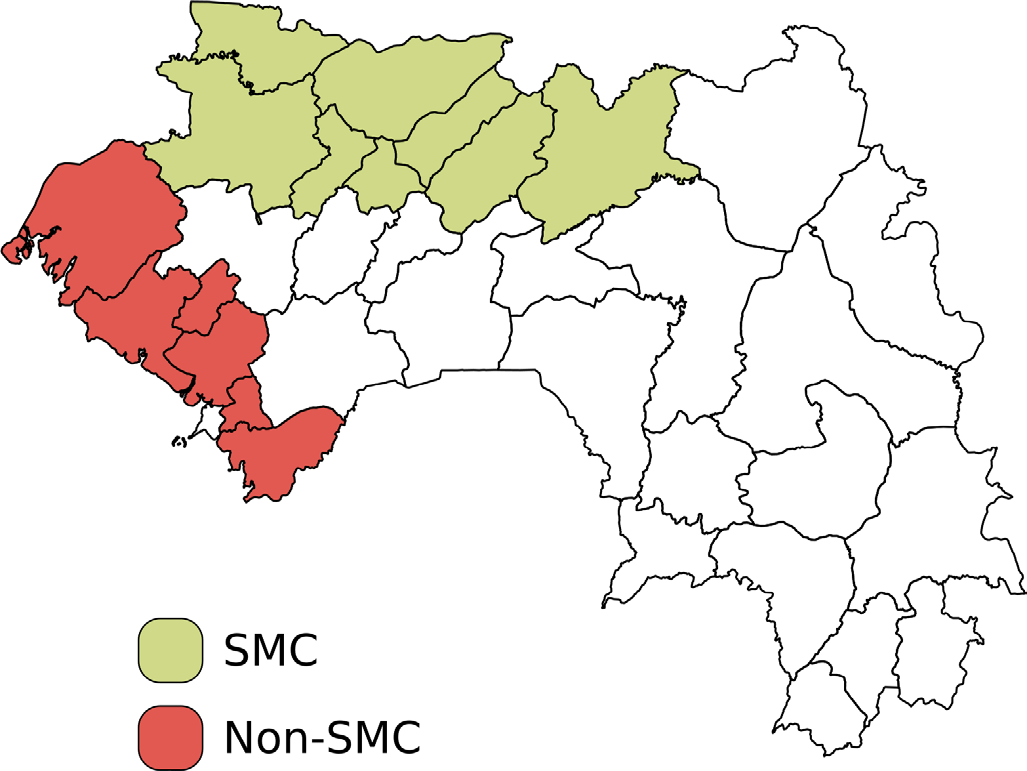
Districts covered by the StopPalu and StopPalu+ in which SMC was performed. Image creator/owner: DB. SMC, seasonal malaria chemoprevention.

Analyses included eight (Labé, Koubia, Tougue, Mali, Lelouma, Gaoual, Koundara and Dinguiraye) and six (Boffa, Boké, Coyah, Dubreka, Forecariyah and Fria) districts where SMC campaigns were or were not performed from January 2014 to November 2021, respectively (figure 1). We adjusted analyses to account for different year of enrolment of districts in the SMC campaign (ie, Labé and Lelouma started SMC in 2017). The 14 study districts were among the districts supported by StopPalu and StopPalu+, projects funded by the US President’s Malaria Initiative and implemented by RTI International. The remaining districts covered by these two projects were the communes of the capital Conakry (ie, Matam, Dixinn, Ratoma, Matoto and Kaloum), which were not eligible for SMC due to their low malaria prevalence; these districts were, therefore, excluded from the analyses.

Among all the study districts’ facilities reporting data to SNIS, we selected those that had a high level of malaria data reporting completeness. Given the long study period and possible difficulties in consistent data reporting, an arbitrary threshold of 10 missing months within the 2014–2021 study period and no more than 3 continuously missing months per year was used to identify facilities with adequate data reporting completeness. We only included public health facilities (ie, hospitals, all health centre types and health posts) supported by StopPalu and StopPalu+. This selection method identified 131 facilities of the 149 in the dataset with adequate reporting completeness. The resulting catchment population based on the selected facilities was 4 451 792 people in 2014, increasing to 5 244 844 people in 2021.

Using the extracted indicator data from SNIS, we calculated the monthly test positivity rate (TPR) and monthly uncomplicated and severe malaria incidence at the district level for the whole population, as well as disaggregated by age groups (<5 years and ≥5 years of age). We used statistical analyses to describe the time trend of the number of tested fevers, TPR, uncomplicated malaria incidence and severe malaria incidence. The changes of these malaria indicators through time were analysed by calculating the CAGR from 2014 to 2021 at the district level. The CAGR was calculated by dividing a time series end value by its beginning value and raising the resulting figure to the inverse number of the time series years subtracting it by one. Thus, for our SMC analyses, the CAGR for each malaria indicator was calculated as follows:

$$CAGR={\left(\frac{value\;2021}{value\;2014}\right)}^{\frac{1}{n}}-1$$

where n is the year of SMC implementation. Testing for differences in malaria indicator values and their CAGR between SMC and non-SMC districts were performed by using the Wilcoxon’s signed rank test.^27^ A full description of the analysis steps is provided in online supplemental file 1.

10.1136/bmjgh-2023-013898.supp1 Supplementary data

The reflexivity statement to promote equitable authorship in the publication of research from international partnerships is appended as online supplemental file 2.

10.1136/bmjgh-2023-013898.supp2 Supplementary data

## Results

### SMC coverage

In the districts included in the analyses, 8.1 million treatment doses of SP+AQ were administered to eligible CU5 between 2015 and 2021, resulting in an average annual programmatic SMC coverage of 89% (range between years: 86%–93%).

### Number of clinical malaria cases tested

**Figure 2:**
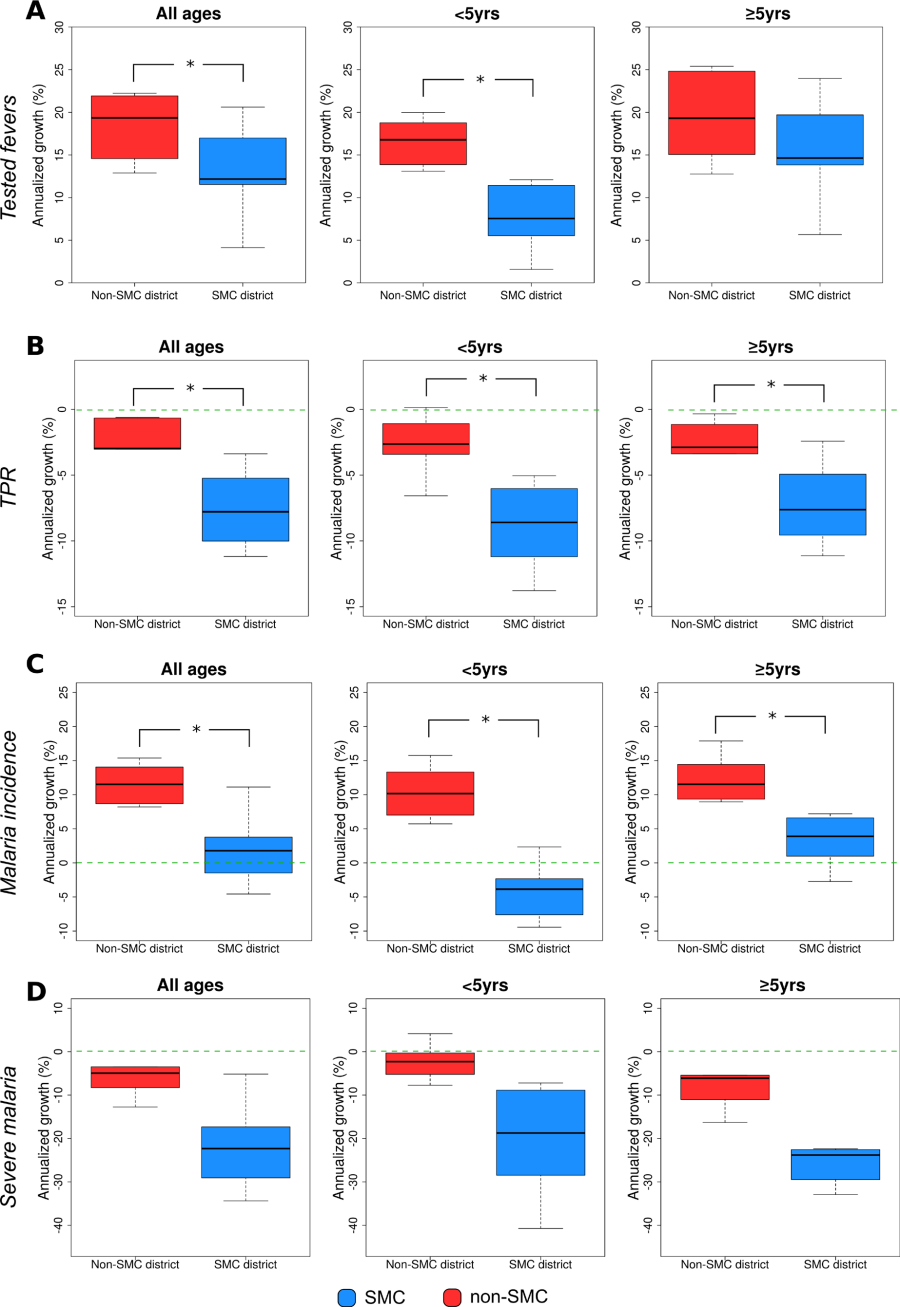
CAGR of tested fevers (A), TPR (B), uncomplicated malaria incidence (C), and severe malaria incidence (D) in SMC districts and non-SMC districts from 2014 to 2021. The ‘*’ symbol represents a significant difference (p<0.05, Wilcoxon’s test) between CAGR values of SMC district and non-SMC districts. Image creator/owner: DB. CAGR, compound annual growth rate; SMC, seasonal malaria chemoprevention; TPR, test positivity rate.

From January 2014 to November 2021, 5 002 551 clinical malaria cases were tested in the health facilities included in the study, of which 1 658 637 were CU5 (33.2%) (table 1). Among all tested clinical cases, 2 484 794 (49.7%) and 2 517 757 (50.3%) were tested in SMC and non-SMC districts, respectively; in both district groups the proportion of clinical malaria in CU5 was approximately 30% of all tested clinical cases (SMC districts=29.8% vs non-SMC districts=36.5%) (table 1). From 2014 to 2021, the trend of tested clinical cases in CU5 and PO5 showed consistent increase in all districts (online supplemental figure S1). The 2014–2021 CAGR of tested clinical cases for SMC districts (median=12.2%, range=4.1%, 20.6%) was significantly lower compared with that of non-SMC districts (median=19.3%; range=12.9%, 22.2 %) (Wilcoxon’s test, p<0.05) (figure 2A). When disaggregated by age, the CAGR of tested clinical cases was lower in SMC districts compared with non-SMC districts for both CU5 and PO5, but it was only statistically significant for CU5 (Wilcoxon’s test, p<0.05) (figure 2A).

**Table 1:** Number of tested fevers, positive tests and positive rate divided by age group and SMC coverage during the study period (January 2014–November 2021)

Age group	All districts		SMC districts		Non-SMC district	
	Tested	Positive (%)	Tested	Positive (%)	Tested	Positive (%)
<5 years age group	1 658 637	915 831 (55.2)	739 403	344 420 (46.6)	919 234	571 411 (62.2)
≥5 years age group	3 343 914	1 928 125 (57.7)	1 745 391	904 818 (51.8)	1 598 523	1 023 307 (64.1)
Total	5 002 551	2 843 956 (56.9)	2 484 794	1 249 238 (50.3)	2 517 757	1 594 718 (63.4)

Image creator/owner: RR.

SMC, seasonal malaria chemoprevention.

### Test positivity rate

**Figure 3:**
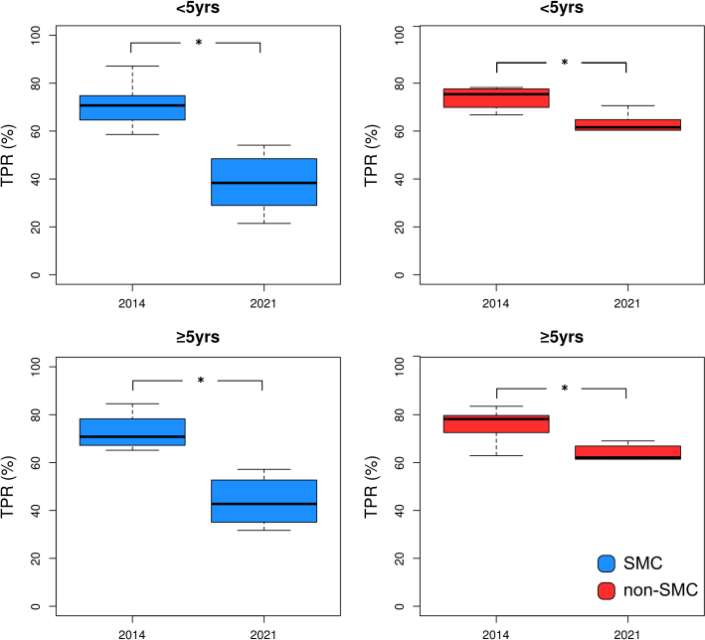
Boxplot comparing TPR of SMC (left column) and non-SMC (right column) districts by age group of 2014 to November 2021 for <5 years and ≥5 years age group. The ‘*’ symbol represents a significant difference (p<0.05, Wilcoxon’s test) between SMC and non-SMC districts. Image creator/owner: DB. SMC, seasonal malaria chemoprevention; TPR, test positivity rate.

The TPR among individuals who were tested from 2014 to 2021 was 56.9% (2 843 956 people), with PO5 having a slightly higher TPR (57.7%) compared with CU5 (55.2%) (table 1). When SMC was accounted for, SMC districts had a lower TPR compared with non-SMC districts for each age group, with the difference in TPR being 15.6 and 12.3 percentage points for CU5 and PO5, respectively (table 1). Comparing the TPR among districts from 2014 to 2021, the TPR steadily declined in SMC districts (online supplemental figure S2A), while the decline in most non-SMC districts stopped in 2019, followed by an increase during 2020 and 2021 (online supplemental figure S2B). The TPR trend during the 2014–2021 study period showed a malaria season characterised by a high transmission season between July and December (Guinea’s rainy season: June–November), followed by a low transmission period occurring between January and May (Guinea’s dry season: December–May) (online supplemental figure S3); peak malaria transmission usually occurred between August and September. The TPR of SMC and non-SMC districts was similar in 2014 (Wilcoxon’s test, p>0.05), but evolved annually to become significantly different in 2021 (Wilcoxon’s test, p<0.05) (online supplemental figure S3). When disaggregated by age, for both age groups, the reduction in TPR between 2014 and 2021 in SMC districts (CU5 tested fevers: median=−46.6%, range=−64.6%, −30.3%; PO5 tested fevers: median=−42.6%, range=−56.2%, −15.8%) was more than two times higher compared with the reduction in non-SMC districts (CU5 tested fevers: median=−17.1%, range=−37.9%, 1.1%; PO5 tested fevers: median=−18.5%, range=−56.2%, −2.8%) (figure 3). The CAGR of TPR of all age groups was statistically lower in the SMC (median=−7.8%, range=−9.7%, −5.5%) compared with non-SMC (median=−3.0%, range=−3.0%, −1.2%) districts (Wilcoxon’s test, p<0.05, figure 2B). When disaggregated by age, the CAGR was significantly different for both CU5 (SMC districts: median=−8.6%, range=−10.9%, −6.3%; non-SMC districts: median=−2.6%, range=−3.3%, −1.4%) and PO5 (SMC districts: median=−7.6%, range=−9.4%, −5.2%; non-SMC districts: median=−2.9%, range=−3.3%, −1.5%) when comparing the two district groups (Wilcoxon’s test, p<0.05, figure 2B).

### Uncomplicated malaria incidence

**Figure 4:**
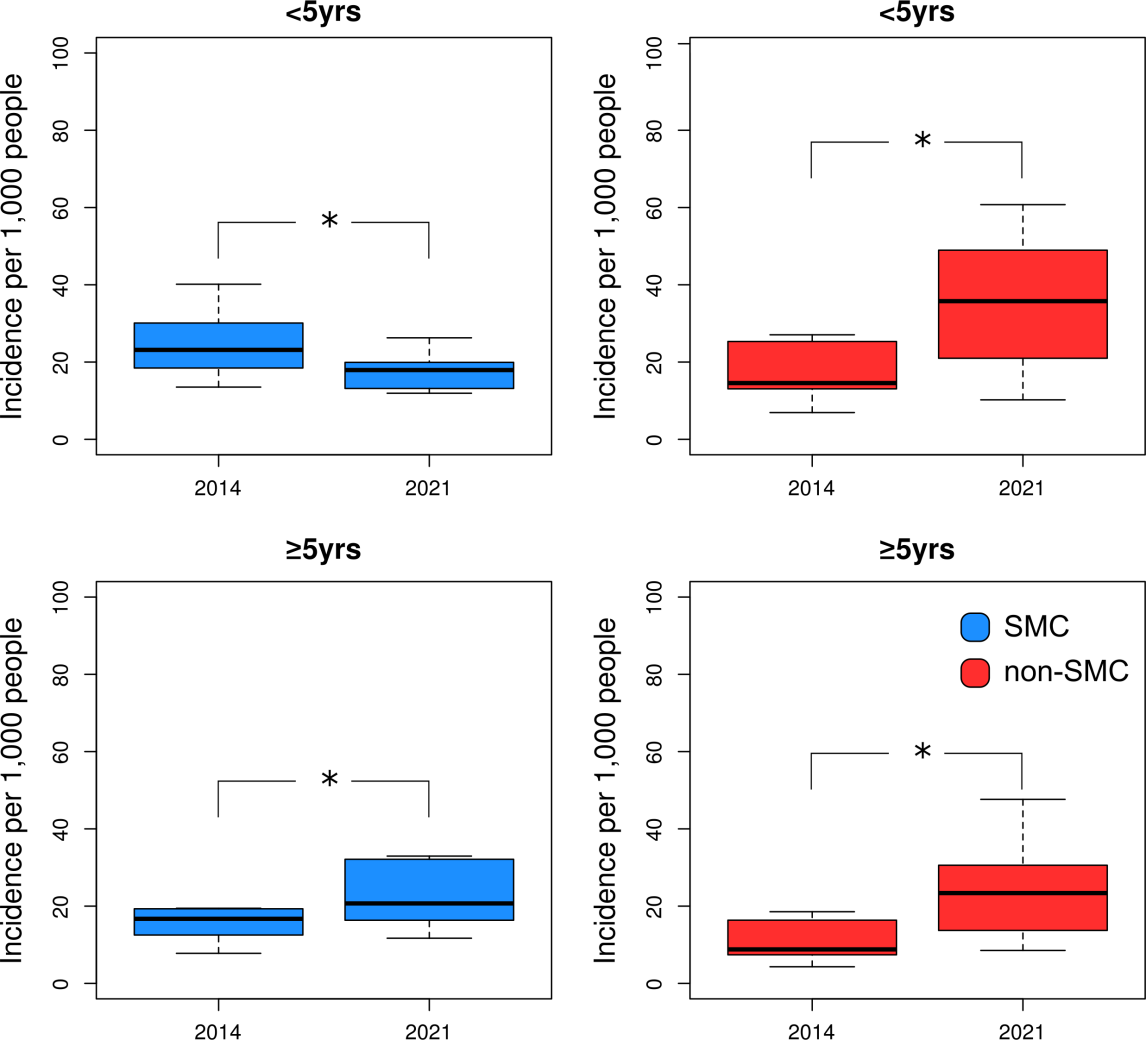
Boxplot comparing uncomplicated malaria incidence of SMC (left column) and non-SMC (right column) districts per age group of 2014 to April 2019 for <5 years age group and ≥5 years age group. The ‘*’ symbol represents a significant difference (p<0.05, Wilcoxon’s test) between SMC and non-SMC districts. Image creator/owner: DB. SMC, seasonal malaria chemoprevention.

The median malaria incidence in SMC districts was 18.5 cases per 1000 people (IQR=14.5–21.6) in 2014 and 19.1 cases per 1000 people (IQR=16.4–28.6) in 2021; the median incidence in non-SMC districts was 10.3 cases per 1000 people (IQR=9.1–17.3) in 2014 and 26.7 cases per 1000 people (IQR=18.2–33.7) in 2021 (online supplemental figure S4). Thus, both SMC and non-SMC districts experienced an increase in incidence from 2014 to 2021. Comparing the incidence trend among the age groups and districts, only the incidence of CU5 in SMC districts showed a declining trend from 2014 to 2021. Incidence was shown to be statistically lower during the low transmission seasons in the SMC districts compared with non-SMC districts (Wilcoxon’s test) (online supplemental figure S5). When disaggregated by age, malaria incidence was only reduced for CU5 between 2014 and 2021 in SMC districts (figure 4). The CAGR in all-age malaria incidence in SMC districts (median=1.8%, range=−0.9%, 3.5%) was significantly lower compared with non-SMC districts (median=11.5%, range=8.8%, 14.0%) (Wilcoxon’s test p<0.05, figure 2C). When disaggregated by age, the CAGR between SMC and non-SMC districts was significantly different for both CU5 (SMC districts: median=−3.9%, range=−7.6%, −2.6%; non-SMC districts: median=10.2%, range=7.5%, −12.9%) and PO5 (SMC districts: median=3.9%, range=1.6%, 6.3%; non-SMC districts: median=11.5%, range=9.6%, 14%) when comparing the two district groups (Wilcoxon’s test, p<0.05, figure 2C).

### Number of severe malaria cases

**Figure 5:**
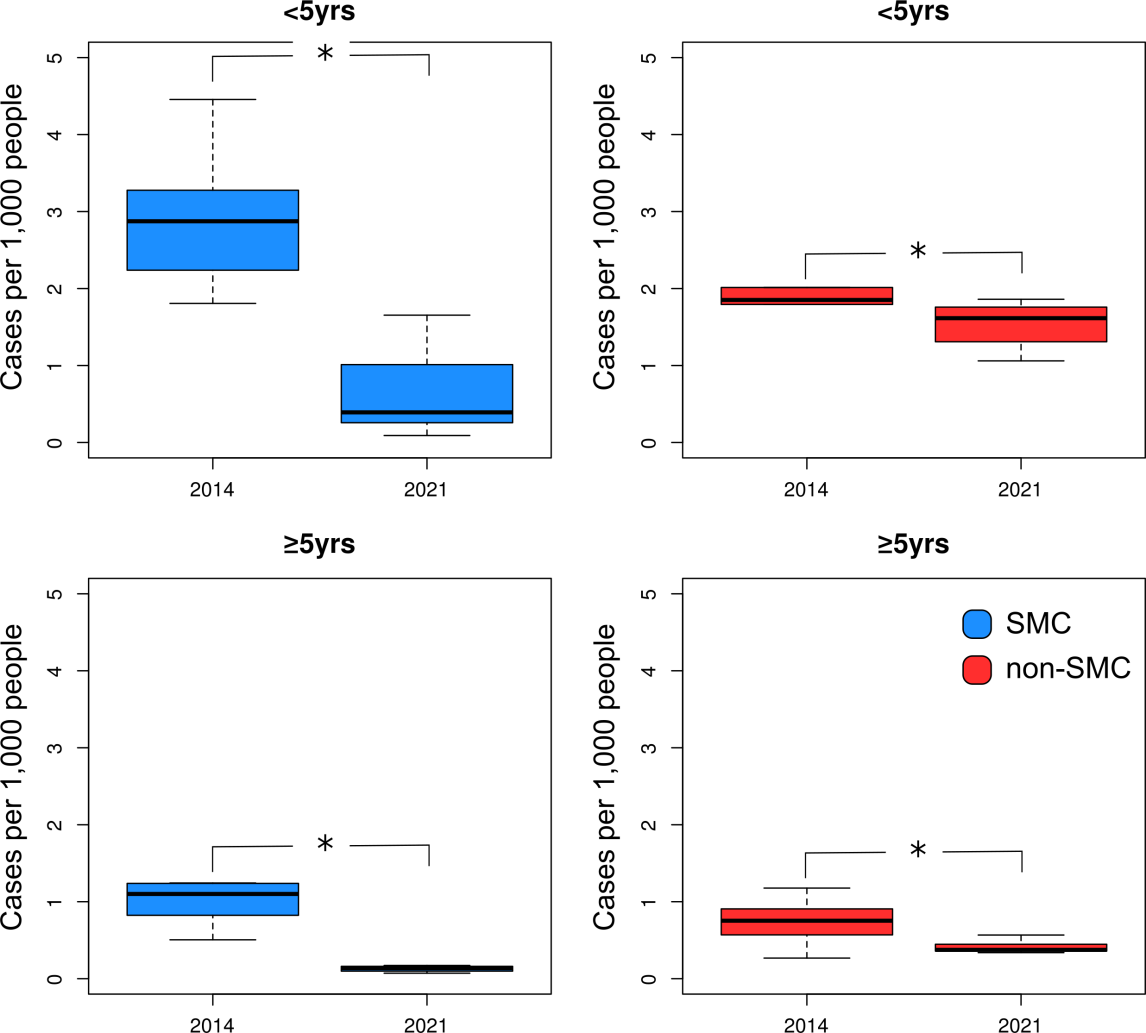
Boxplot comparing severe malaria cases per 1000 people in SMC (left column) and non-SMC (right column) districts per age group of 2014 to April 2019 <5 yrs age group and ≥5 years age group. The ‘*’ symbol represents a significant difference (p<0.05, Wilcoxon’s test) between SMC district and non-SMC districts. Image creator/owner: DB. SMC, seasonal malaria chemoprevention.

From January 2014 to November 2021, 264 726 severe malaria cases were reported from the facilities included in the study, of which 111 647 (42.2%) were from SMC districts and 153 079 (57.8%) from non-SMC districts. The proportion of severe malaria in CU5 was similar in SMC districts (40 426 cases, 36.2%) and non-SMC districts (56 459 cases, 36.8%). The median severe malaria incidence in SMC districts declined from 1.4 cases per 1000 people (IQR=1.1–1.6) in 2014 to 0.2 cases per 1000 people (IQR=0.1–0.3) in 2021, and from 0.9 cases per 1000 people (IQR=0.8–1.1) to 0.6 cases per 1000 people (IQR=0.5–0.7) in non-SMC districts (online supplemental figure S6). The incidence of severe cases was higher in PO5 in both SMC districts and non-SMC districts. The reduction of severe malaria cases declined in both age groups but was significantly higher in CU5 (Wilcoxon’s test p<0.05) (figure 5). The CAGR of severe malaria incidence showed that all age groups experienced a decline in severe malaria in both SMC and non-SMC districts. However, this decline was significantly higher in SMC (median=−22.3%, range=−27.6%, −18.2%) than in non-SMC (median=−5.1%, range=−7.7, –3.6) districts for the entire population, as well as both CU5 and PO5 (Wilcoxon’s test p<0.05) (figure 2D).

## Discussion

Randomised controlled trials have shown that SMC can reduce the incidence of clinical malaria, parasitaemia, anaemia, severe malaria and all-cause mortality in CU5, when effectively deployed.^4–7^ Several studies have analysed the effectiveness of SMC when implemented at large operational scales, showing SMC protective effectiveness against clinical malaria ranging from 73–98% in CU5 at 28 and 42-days post-SMC administration, respectively.^28 29^ However, these studies relied on large samples of at-risk populations (ie, CU5), either following case/control study designs,^28 29^ or using nationally representative surveys such as Demographic and Health Surveys or Malaria Indicator Surveys.^30 31^ While such approaches are certainly robust, they can be onerous, time-consuming, and costly, and may not allow for continuous monitoring of the intervention across all SMC implementation areas.

As countries’ HMIS are strengthened, there is the opportunity to increasingly use data that is routinely (passively) collected by health facilities and reported by districts.^32–36^ Using district-level data from Guinea’s HMIS collected between 2014 and 2021, we show the programmatic impact of SMC on malaria trends in targeted districts, confirming the effectiveness of SMC shown in various controlled research studies. Our analyses show that—in a context of strengthened case management and medium to high coverage of LLINs—SMC significantly reduced the TPR, uncomplicated malaria incidence, and the incidence of severe malaria in CU5 in Guinea between 2014 and 2021. These findings also corroborate other recent analyses estimating SMC effectiveness through use of routine health information system data. Thus, in Chad, using generalised additive modelling, Richardson *et al* 37 estimated that SMC reduced the number of suspected and confirmed malaria in CU5 by 18% (95% CI 6% to 28%) and 19% (95% CI 7% to 29%) at primary health facilities in 23 health districts during the months of SMC implementation, respectively. Similarly, an evaluation of the ACCESS-SMC programme using a difference-in-differences approach of DHIS2 data observed a 45.0% and 55.2% reduction in malaria outpatients after 2 years of SMC implementation in Burkina Faso and The Gambia, respectively; the study also reported substantial reductions in the number of malaria inpatients and malaria hospital deaths following SMC.^29^

Interestingly, we show that the effect of SMC is particularly pronounced in the dry season rather than the rainy season. We hypothesise that SMC results in CU5 either being fully protected from infection or, if infected, only having infections with low parasitaemia (which may be due to incomplete adherence to the 3-day regimen or drug resistance).^38^ Combined with the lower abundance and density of mosquitoes during the dry season, this then results in lower transmission rates, which leads to a lower incidence. Similar observations have been made in studies evaluating vector control interventions.^39^ These observations do show, however, that in Guinea SMC should possibly start earlier than in July (ie, to have a greater effect on infection protection and transmission intensity during peak transmission season), and that studies monitoring SMC impact should potentially monitor effectiveness beyond the currently recommended 28–42 days post-SMC administration.^40^

Moreover, unlike prior controlled academic research and other studies, our analyses also demonstrate an effect of SMC administered to CU5, although lower, on PO5, specifically for TPR, uncomplicated malaria incidence, and severe malaria incidence. Such effect seems plausible, given that—even in a context of decreasing malaria prevalence such as Guinea^1^ —CU5 still represent a large proportion of all cases (ie, 55.2% for the 2014–2021 study period; table 1) and, thus, a substantial proportion of the human reservoir infecting anopheline mosquitoes. Any significant intervention effect reducing the CU5 *Plasmodium* reservoir will lower transmission intensity and, thus, spill over and reduce malariometric indicators in PO5.

As support continues for malaria elimination and additional means for malaria control are introduced (eg, vaccines), alternative methods for measuring impact must be explored outside trial settings as controlled and observational research studies to investigate effectiveness at scale are very costly. Multiple impact evaluations and approaches that address gaps or biases in data and triangulating between data sources strengthens the plausibility of programme impact. We used the CGAR as an analytical method. The CAGR is a commonly used methodology in the field of economics to describe changes in (economic) growth from the beginning to the end of a time series. The advantage of using CAGR compared with other analytical approaches—such as average annualised rates—is that the resulting estimate is not affected by fluctuations within the time series that could produce misleading results. The CAGR methodology has previously been used in the public health field to evaluate healthcare systems and effects of interventions through time.^41–43^ An additional advantage of the approach is that, because it is algebra based, it does not require advanced data analytics or modelling expertise, and thus can be routinely applied by programme personnel with a basic understanding of calculus (eg, in MS Excel worksheets or even DHIS2).

Because SMC is consistently proving to be a high coverage and effective intervention, several countries with seasonal malaria transmission outside of the Sahel are now piloting SMC to be included in their malaria intervention package (eg, Uganda^44^ and Mozambique^45^). WHO recently amended the SMC guidelines to give countries flexibility to change the SMC regimen (eg, by adding additional SMC cycles to cover longer peak transmission seasons, or by including older age groups).^8^ Such step is likely going to increase the effect of SMC even further, either in CU5 (if additional SMC cycles are added) or in older age groups (if older age groups are covered with SMC)—indeed recent studies in Burkina Faso, Mali and Senegal seem to corroborate this.^46–48^ Moreover, there is increased interest to use SMC campaigns to integrate or leverage other health interventions,^49–51^ the rationale being that (1) other health interventions that may not have as high population acceptance or coverage as SMC would benefit from the high acceptance and coverage of SMC and (2) cost-efficiencies would be obtained, since combining intervention planning, implementation and monitoring would result in various economies of scale (eg, training of health workers, transport costs, limiting health workers’ time to implement campaigns rather than providing health services).

### Limitations

Several potential caveats of our analyses should be highlighted. First, our analyses did not adjust for intervention coverage, either for SMC or other malaria interventions such as LLINs. Thus, we assume that programmatic coverage, use of and adherence to these interventions is relatively homogenous and that the only difference in overall malaria intervention coverage between the two sets of districts included in our analyses is the administration of SMC to CU5. Post-SMC round assessments consistently showed programmatic coverage of 82%–93% across districts included in our analyses. Moreover, as per 2021 Malaria Indicator Survey data,^52^ LLIN use in CU5 in households with at least 1 LLINs was 60.4% in survey enumeration areas in SMC districts, compared with 65.6% for non-SMC districts.

Second, using routine HMIS data for impact evaluations can be problematic due to internal validity, completeness and potential bias in estimates of effect, and caution must be exercised in interpreting analytical findings.^32^ Additionally, challenges with HMIS data are that they are dependent on the proportion of infected individual cases seeking treatment for malaria at public sector health facilities; the proportion of patients seeking care who are parasitologically diagnosed; and facilities reporting all suspected and confirmed malaria cases consistently over time. Thus, for example, even though SMC was implemented in Guinea in 2015, an increase in the number of fevers was observed in CU5, suggesting a lack of impact of SMC. However, during this period, changes in access to health services occurred, such as improvement in malaria case detection and diagnosis at facility and community levels, removal of user access fees for CU5 and pregnant women, and improvement in the availability of malaria diagnostic and case management commodities. We controlled for such bias by excluding health facilities with incomplete reporting (ie, facilities with 10 months of missing data throughout the study period, and no more than three continuously missing months per year), and noted that these facilities were evenly spread across SMC and non-SMC districts. We also note that our analyses of the SMC effect in CU5 was consistently observed across all malariometric indicators included in our analysed.

Third, malaria testing to identify cases in Guinea is largely RDT based. Although used tests have a high sensitivity and specificity, it is possible that low parasitaemic and asymptomatic infections were missed. It is unlikely, however, that such infections would have clustered in a specific spatiotemporal pattern across SMC compared with non-SMC districts, and, thus, would change our findings and conclusions.

Fourth, we did not adjust for climate confounders that are known to possibly affect malaria transmission, intervention implementation or health service access, such as temperature and rainfall. Given the moderate distances between SMC and non-SMC districts, we assumed that changes in temperature and rainfall pattern would likely affect both sets of districts similarly. Outlined CGAR approach certainly represents a simple approach comparing malaria trends between districts experiencing differing variability in climate variables,

Fifth, we delineated our analyses to district boundaries, rather than a defined area size (eg, 25 km^2^). This was done because routinely collected HMIS data gets aggregated at that level and districts are the lowest administrative unit in Guinea that plan, implement and monitor malaria programming; any response to an increase in malaria cases would occur at district level.

Finally, spatial analyses performed using arbitrary spatial divisions such as district administrative boundaries can be affected by the modifiable areal unit problem.^53 54^ For example, cases that were reported at a health facility in a given district could stem from households in a different district, and therefore, bias district-level malaria HMIS data. Thus, the possibility remains that cases got infected outside of their district (eg, during travel).

## Conclusion

Our study provides evidence to support that—even in an operational programming context—adding SMC to the comprehensive package of malaria interventions yields a positive epidemiological impact and results in a greater reduction in TPR, malaria incidence and hospitalisations in CU5 than when implementing an intervention package without SMC. We also show that the use of routine HMIS data represents a viable method for assessing the epidemiological impact of public health interventions in the absence of trial studies, household surveys and extensive covariate data.

## Data Availability

Data are available on reasonable request. The data analysed originated from Guinea’s routine health management information system; data may be available on reasonable request from Guinea’s National Malaria Control Programme.

## References

[1] Ministry of Health . Annual bulletin of the National Malaria Control Program 2020. Conakry Ministry of Health; 2021.

[2] NMCP . National Malaria Strategic Plan 2018–2022. Conakry Ministry of Health; 2018.

[3] WHO . Seasonal malaria chemoprevention for Plasmodium falciparum malaria control in highly seasonal transmission areas of the Sahel sub-region in Africa. Geneva WHO; 2012.

[4] Wilson AL , IPTc Taskforce IP . A systematic review and meta-analysis of the efficacy and safety of intermittent preventive treatment of malaria in children (IPTc). PLoS One 2011;6:e16976. 10.1371/journal.pone.0016976 21340029 PMC3038871

[5] Meremikwu MM , Donegan S , Sinclair D , et al . Intermittent preventive treatment for malaria in children living in areas with seasonal transmission. Cochrane Database Syst Rev 2012;2012:CD003756. 10.1002/14651858.CD003756.pub4 22336792 PMC6532713

[6] Cairns M , Roca-Feltrer A , Garske T , et al . Estimating the potential public health impact of seasonal malaria chemoprevention in African children. Nature 2012;3:881. 10.1038/ncomms1879 PMC3621394 22673908

[7] WHO . Guidelines for malaria. Geneva WHO; 2022. Available: https://www.who.int/teams/global-malaria-programme/guidelines-for-malaria

[8] WHO . Updated WHO recommendations for malaria chemoprevention among children and pregnant women. Geneva WHO; 2022. Available: https://www.who.int/news/item/03-06-2022-Updated-WHO-recommendations-for-malaria-chemoprevention-among-children-and-pregnant-women

[9] Dicko A , Diallo AI , Tembine I , et al . Intermittent preventive treatment of malaria provides substantial protection against malaria in children already protected by an insecticide-treated bednet in Mali: a randomised, double-blind, placebo-controlled trial. PLoS Med 2011;8:e1000407. 10.1371/journal.pmed.1000407 21304923 PMC3032550

[10] Konaté AT , Yaro JB , Ouédraogo AZ , et al . Intermittent preventive treatment of malaria provides substantial protection against malaria in children already protected by an insecticide-treated bednet in Burkina Faso: a randomised, double-blind, placebo-controlled trial. PLoS Med 2011;8:e1000408. 10.1371/journal.pmed.1000408 21304925 PMC3032552

[11] Cissé B , Sokhna C , Boulanger D , et al . Seasonal intermittent preventive treatment with artesunate and sulfadoxine-pyrimethamine for prevention of malaria in Senegalese children: a randomised, placebo-controlled, double-blind trial. Lancet 2006;367:659–67. 10.1016/S0140-6736(06)68264-0 16503464

[12] Diawara F , Steinhardt LC , Mahamar A , et al . Measuring the impact of seasonal malaria chemoprevention as part of routine malaria control in Kita, Mali. Malar J 2017;16:325. 10.1186/s12936-017-1974-x 28797263 PMC5553795

[13] Kweku M , Liu D , Adjuik M , et al . Seasonal intermittent preventive treatment for the prevention of anaemia and malaria in Ghanaian children: a randomized, placebo controlled trial. PLoS One 2008;3:e4000. 10.1371/journal.pone.0004000 19098989 PMC2602973

[14] Ntab B , Cissé B , Boulanger D , et al . Impact of intermittent preventive anti-malarial treatment on the growth and nutritional status of preschool children in rural Senegal (West Africa). Am J Trop Med Hyg 2007;77:411–7. 17827352 PMC3749810

[15] Sokhna C , Cissé B , Bâ EH , et al . A trial of the efficacy, safety and impact on drug resistance of four drug regimens for seasonal intermittent preventive treatment for malaria in Senegalese children. PLoS One 2008;3:e1471. 10.1371/journal.pone.0001471 18213379 PMC2198946

[16] Bojang K , Akor F , Bittaye O , et al . A randomised trial to compare the safety, tolerability and efficacy of three drug combinations for intermittent preventive treatment in children. PLoS One 2010;5:e11225. 10.1371/journal.pone.0011225 20574538 PMC2888611

[17] Zongo I , Milligan P , Compaore YD , et al . Randomized noninferiority trial of dihydroartemisinin-piperaquine compared with sulfadoxine-pyrimethamine plus amodiaquine for seasonal malaria chemoprevention in Burkina Faso. Antimicrob Agents Chemother 2015;59:4387–96. 10.1128/AAC.04923-14 25918149 PMC4505196

[18] Patouillard E , Conteh L , Webster J , et al . Coverage, adherence and costs of intermittent preventive treatment of malaria in children employing different delivery strategies in Jasikan, Ghana. PLoS One 2011;6:e24871. 10.1371/journal.pone.0024871 22073137 PMC3207811

[19] Bojang KA , Akor F , Conteh L , et al . Two strategies for the delivery of IPTc in an area of seasonal malaria transmission in the Gambia: a randomised controlled trial. PLoS Med 2011;8:e1000409. 10.1371/journal.pmed.1000409 21304921 PMC3032548

[20] Buffet PA , Briand V , Rénia L , et al . Intermittent preventive antimalarial treatment to children (IPTc): firebreak or fire trap? Trends Parasitol 2008;24:482–5; 10.1016/j.pt.2008.07.007 18782680

[21] Beeson JG , Rogerson SJ , Mueller I , et al . Intermittent preventive treatment to reduce the burden of malaria in children: new evidence on integration and delivery. PLoS Med 2011;8:e1000410. 10.1371/journal.pmed.1000410 21304919 PMC3032544

[22] Barreto ML . Efficacy, effectiveness, and the evaluation of public health interventions. J Epidemiol Community Health 2005;59:345–6. 10.1136/jech.2004.020784 15831679 PMC1733082

[23] The malERA Refresh Consultative Panel on Health Systems and Policy Research. malERA: an updated research agenda for health systems and policy research in malaria elimination and eradication. PLoS Medicine 2017;14:e1002454. 10.1371/journal.pmed.1002454 29190289 PMC5708613

[24] Beavogui AH , Delamou A , Camara BS , et al . Prevalence of malaria and factors associated with infection in children aged 6 months to 9 years in Guinea: results from a national cross-sectional study. Parasite Epidemiol Control 2020;11:e00162. 10.1016/j.parepi.2020.e00162 32715113 PMC7378695

[25] Sayre D , Camara A , Barry Y , et al . Combined epidemiologic and entomologic survey to detect urban malaria transmission, Guinea, 2018. Emerg Infect Dis 2021;27:599–602. 10.3201/eid2702.191701 33496219 PMC7853535

[26] Reynolds E , Martel LD , Bah MO , et al . Implementation of DHIS2 for disease surveillance in Guinea: 2015-2020. Front Public Health 2021;9:761196. 10.3389/fpubh.2021.761196 35127614 PMC8811041

[27] Hollander M , Wolfe DA . Nonparametric statistical methods. New York John Wiley & Sons; 1973.

[28] Cairns M , Ceesay SJ , Sagara I , et al . Effectiveness of seasonal malaria chemoprevention (SMC) treatments when SMC is implemented at scale: case-control studies in 5 countries. PLoS Med 2021;18:e1003727. 10.1371/journal.pmed.1003727 34495978 PMC8457484

[29] ACCESS-SMC Partnership . Effectiveness of seasonal malaria Chemoprevention at scale in West and central Africa: an observational study. Lancet 2020;396:1829–40. 10.1016/S0140-6736(20)32227-3 33278936 PMC7718580

[30] Druetz T . Evaluation of direct and indirect effects of seasonal malaria chemoprevention in Mali. Sci Rep 2018;8:8104. 10.1038/s41598-018-26474-6 29802375 PMC5970148

[31] de Cola MA , Sawadogo B , Richardson S , et al . Impact of seasonal malaria chemoprevention on prevalence of malaria infection in malaria indicator surveys in Burkina Faso and Nigeria. BMJ Glob Health 2022;7:e008021. 10.1136/bmjgh-2021-008021 PMC9121431 35589153

[32] Rowe AK , Kachur SP , Yoon SS , et al . Caution is required when using health facility-based data to evaluate the health impact of malaria control efforts in Africa. Malar J 2009;8:1–3. 10.1186/1475-2875-8-209 19728880 PMC2743707

[33] Afrane YA , Zhou G , Githeko AK , et al . Utility of health facility-based malaria data for malaria surveillance. PLoS One 2013;8:e54305. 10.1371/journal.pone.0054305 23418427 PMC3572108

[34] Ashton RA , Bennett A , Yukich J , et al . Methodological considerations for use of routine health information system data to evaluate malaria program impact in an era of declining malaria transmission. Am J Trop Med Hyg 2017;97:46–57. 10.4269/ajtmh.16-0734 28990915 PMC5619932

[35] WHO . Analysis and use of health facility data: guidance for malaria programme managers. Geneva World Health Organization; 2018.

[36] Wagenaar BH , Sherr K , Fernandes Q , et al . Using routine health information systems for well-designed health evaluations in low- and middle-income countries. Health Policy Plan 2016;31:129–35. 10.1093/heapol/czv029 25887561 PMC4751224

[37] Richardson S , Moukenet A , Diar MSI , et al . Modeled impact of seasonal malaria chemoprevention on district-level suspected and confirmed malaria cases in Chad based on routine clinical data (2013-2018). Am J Trop Med Hyg 2021;105:1712–21. 10.4269/ajtmh.21-0314 34662864 PMC8641328

[38] Chotsiri P , White NJ , Tarning J . Pharmacokinetic considerations in seasonal malaria chemoprevention. Trends Parasitol 2022;38:673–82. 10.1016/j.pt.2022.05.003 35688778

[39] Fullman N , Burstein R , Lim SS , et al . Nets, spray or both? The effectiveness of insecticide-treated nets and indoor residual spraying in reducing malaria morbidity and child mortality in sub-Saharan Africa. Malar J 2013;12:62. 10.1186/1475-2875-12-62 23402342 PMC3610288

[40] WHO . Malaria chemoprevention efficacy study protocol. Geneva World Health Organization; 2022.

[41] Hwang J , Shen JJ , Kim SJ , et al . Opioid use disorders and hospital palliative care among patients with gastrointestinal cancers: Ten-year trend and associated factors in the U.S. from 2005 to 2014. Medicine (Baltimore) 2020;99:e20723. 10.1097/MD.0000000000020723 32569209 PMC7310906

[42] Joo MK , Yoo JW , Mojtahedi Z , et al . Ten-year trends of utilizing palliative care and palliative procedures in patients with gastric cancer in the United States from 2009 to 2018 - a nationwide database study. BMC Health Serv Res 2022;22:20. 10.1186/s12913-021-07404-1 34980097 PMC8725552

[43] Moses H , Matheson DHM , Dorsey ER , et al . The anatomy of health care in the United States. JAMA 2013;310:1947. 10.1001/jama.2013.281425 24219951

[44] Nuwa A , Baker K , Bonnington C , et al . A non-randomized controlled trial to assess the protective effect of SMC in the context of high parasite resistance in Uganda. Malar J 2023;22:63. 10.1186/s12936-023-04488-4 36814301 PMC9945593

[45] Baker K , Aide P , Bonnington CA , et al . Feasibility, acceptability, and protective efficacy of seasonal malaria chemoprevention implementation in Nampula province, Mozambique: protocol for a hybrid effectiveness-implementation study. JMIR Res Protoc 2022;11:e36403. 10.2196/36403 36149743 PMC9547334

[46] Cissé B , Ba EH , Sokhna C , et al . Effectiveness of seasonal malaria chemoprevention in children under ten years of age in Senegal: a stepped-wedge cluster-randomised trial. PLoS Med 2016;13:e1002175. 10.1371/journal.pmed.1002175 27875528 PMC5119693

[47] Ndiaye JLA , Ndiaye Y , Ba MS , et al . Seasonal malaria chemoprevention combined with community case management of malaria in children under 10 years of age, over 5 months, in south-east Senegal: a cluster-randomised trial. PLoS Med 2019;16:e1002762. 10.1371/journal.pmed.1002762 30865632 PMC6415816

[48] Bâ EH , Pitt C , Dial Y , et al . Implementation, coverage and equity of large-scale door-to-door delivery of seasonal malaria chemoprevention (SMC) to children under 10 in Senegal. Sci Rep 2018;8:5489. 10.1038/s41598-018-23878-2 29615763 PMC5882955

[49] Bazant E , McPhillips-Tangum C , Shrestha SD , et al . Promising practices for the collaborative planning of integrated health campaigns from a synthesis of case studies. BMJ Glob Health 2022;7:e010321. 10.1136/bmjgh-2022-010321 PMC9756207 36517112

[50] Oresanya O , Phillips A , Okereke E , et al . Co-implementing vitamin A supplementation with seasonal malaria chemoprevention in Sokoto State, Nigeria: a feasibility and acceptability study. BMC Health Serv Res 2022;22:871. 10.1186/s12913-022-08264-z 35791014 PMC9258179

[51] Keita MS , Camara A , Daffe M , et al . Integrating nutrition assessments and seasonal malaria chemoprevention: results from a mixed-methods feasibility study in Guinea. Trop Med Int Health 2023;28:571–5. 10.1111/tmi.13897 37258746

[52] Institut National de la Statistique (INS) and ICF . Malaria indicator survey 2021. Rockville, Maryland, USA INS and ICF; 2021.

[53] Wong DW . The Modifiable areal unit problem (MAUP). In: WorldMinds: geographical perspectives on 100 problems. Dordrecht: Springer, 2004. 10.1007/978-1-4020-2352-1

[54] Alegana VA , Atkinson PM , Wright JA , et al . Estimation of malaria incidence in northern Namibia in 2009 using Bayesian conditional-autoregressive spatial-temporal models. Spat Spatiotemporal Epidemiol 2013;7:25–36. 10.1016/j.sste.2013.09.001 24238079 PMC3839406

