# Supplementary material for: Malaria trends in districts that were targeted and not-targeted for seasonal malaria chemoprevention in children under 5 years of age in Guinea, 2014–2021
Source: BMJ Glob Health. 2024 Feb 26;9(2):e013898. doi: 10.1136/bmjgh-2023-013898 (PMC10900330; doi:10.1136/bmjgh-2023-013898)
Supplement: Supplementary data [file bmjgh-2023-013898supp001.pdf]

Supplementary Information File 1

Bisanzio D, et al. Malaria trends in districts that were targeted and not-targeted for seasonal malaria chemoprevention in children under five years of age in Guinea, 2014–2021

Below is a step-by-step description of the analytical approach to assess malaria trends in districts that were targeted and not-targeted for seasonal malaria chemoprevention in children under five years of age in Guinea, 2014–2021. Analysis of the data can be easily performed in Microsoft Excel or by using any data analysis software (R, STATA, SPSS).

1. Monthly numbers of tested clinical malaria cases, confirmed positive clinical cases, and severe malaria are disaggregated by age group (children <5 years of age [CU5], people ≥5 years of ages [PO5]) were obtained from Guinea's health management information system (HMIS) for public health facilities supported by StopPalu and StopPalu+ in the selected districts.
2. Data on population in the facility catchment areas were obtained from Guinea's HMIS.
3. Health facility HMIS reporting completeness was calculated for each facility to select those facilities with less than 10 missing months of data within the 2014–2021 study period, with no more than 3 continuously missing months per year.
4. Malariometric indicators for each age group and in each district were calculated as follows:
    a. Tested fevers: total number of fevers tested in the district's facilities.
    b. *Test positivity rate (TPR)*: dividing number of malaria-positive fever cases by the total number of tested fever cases.
    c. *Incidence of uncomplicated malaria cases*: number of confirmed malaria cases divided by the population in the district facilities' catchment area; the result was multiplied by 1,000 to obtain number of incident confirmed malaria cases per 1,000 people.
    d. *Incidence of severe malaria cases*: number of severe malaria cases divided by the the population in the district facilities' catchment area; the result was multiplied by 1,000 to obtain number of incident severe malaria cases per 1,000 people.
5. The compound annual growth rate (CAGR) from 2014 to 2021 for tested fevers, TPR, uncomplicated and severe malaria incidence was calculated using the following formula:

   $$CAGR = \left(\frac{value\ 2021}{value\ 2014}\right)^{\frac{1}{n}} - 1$$

   where value 2014 and value 2021 is the value of a given malaria indicator in 2014 and 2021, and n is the number of years from 2014 to 2021.
6. Statistical differences in malariometric indicator values and their CAGR between SMC and non-SMC districts were performed using the Wilcoxon's signed rank test.

Figure S1. Number of tested fevers in SMC and non-SMC districts per age group and year. Plots show tested fevers for all-age groups, <5yrs age group, and ≥5yrs age group in SMC (A) and non-SMC districts (B)

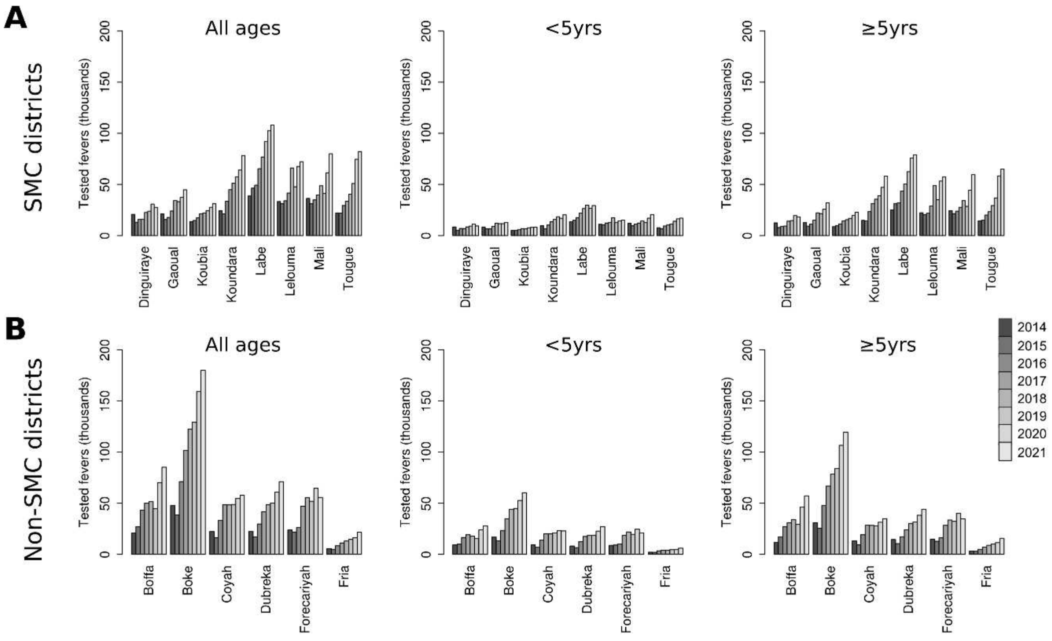

Image creator/owner: Donal Bisanzio

Figure S2. TPR of tested fevers in SMC districts and non-SMC districts per age group and year. Plots show TPR for all-age groups, <5yrs age group, and ≥5yrs age group in SMC (A) and non-SMC districts (B)

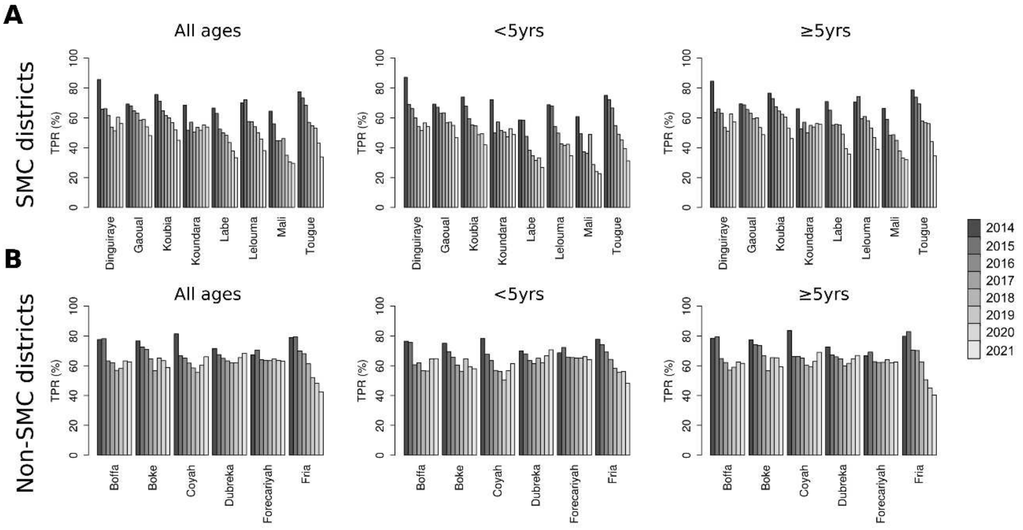

Image creator/owner: Donal Bisanzio

Figure S3. Boxplot of annual TPR of SMC districts and non-SMC districts from January 2014 to November 2021 for all age groups, <5 age group, and ≥5 age group, by year.

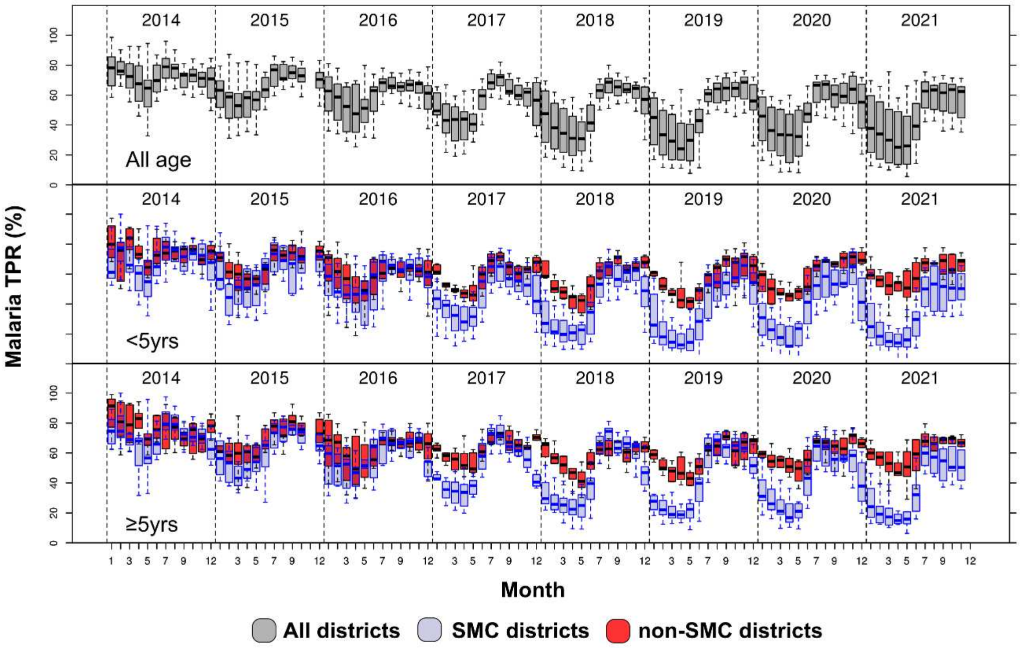

Image creator/owner: Donal Bisanzio

Figure S4. Malaria incidence in SMC (A) and non-SMC (B) districts per age group and year. Plots show malaria incidence for all-age groups, <5yrs age group, and ≥5yrs age group in SMC (A) and non-SMC districts (B).

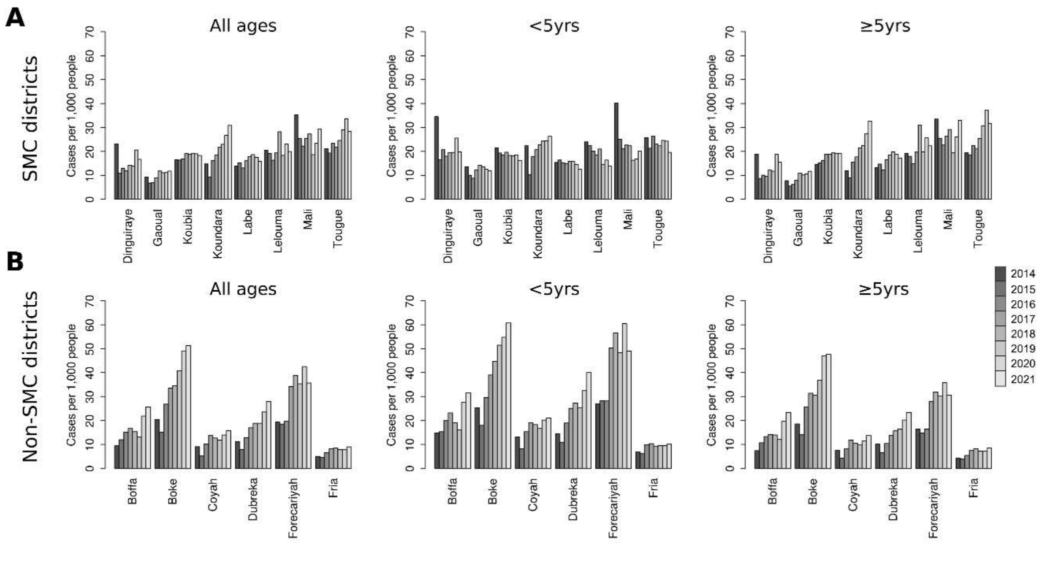

Image creator/owner: Donal Bisanzio

Figure S5. Boxplot of malaria incidence of SMC districts and non-SMC districts from January 2014 to November 2021 for all age groups, <5 yrs age group, and ≥5 yrs age group, by year.

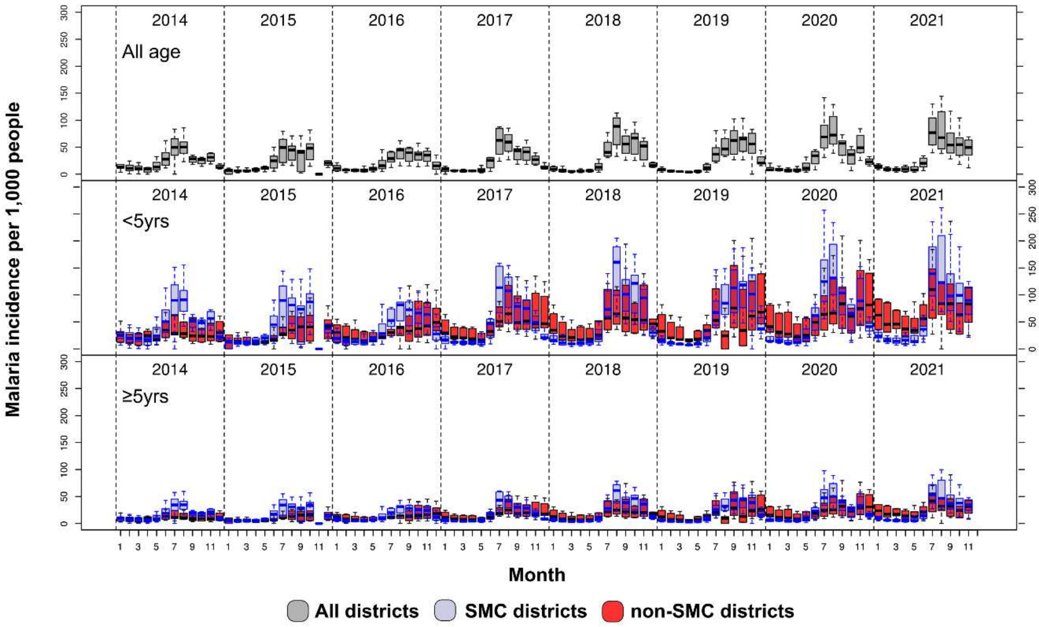

Image creator/owner: Donal Bisanzio

Figure S6. Incidence of severe malaria cases in SMC (A) and non-SMC (B) districts per age group and year. Plots show severe malaria incidence for all-age groups, <5yrs age group, and ≥5yrs age group in SMC (A) and non-SMC districts (B)

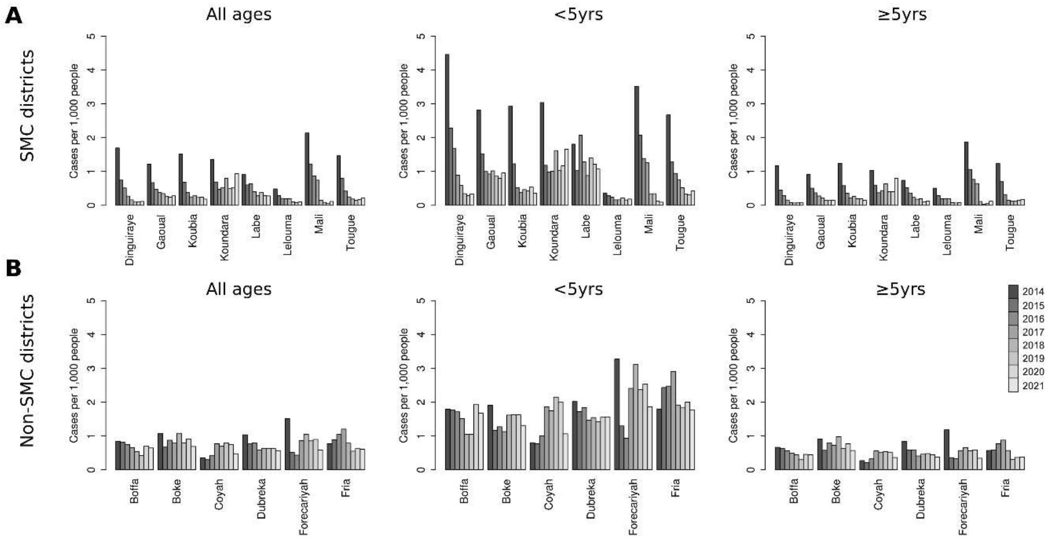

Image creator/owner: Donal Bisanzio
