# Supplementary material for: Malaria trends in districts that were targeted and not-targeted for seasonal malaria chemoprevention in children under 5 years of age in Guinea, 2014–2021
Source: BMJ Glob Health. 2024 Feb 26;9(2):e013898. doi: 10.1136/bmjgh-2023-013898 (PMC10900330; doi:10.1136/bmjgh-2023-013898)
Supplement: Supplementary data [file bmjgh-2023-013898supp002.pdf]

Supplementary Information File 2

Bisanzio D et al. Malaria trends in districts that were targeted and not-targeted for seasonal malaria chemoprevention in children under five years of age in Guinea, 2014–2021.

AUTHOR REFLEXIVITY STATEMENT

1. How does this study address local research and policy priorities?

Seasonal malaria chemoprevention (SMC) is a critical intervention in the prevention and control of malaria in West Africa, including Guinea. SMC can reduce the incidence of clinical malaria in the 28 days following administration by up to 75% when effectively deployed. While there is strong evidence of the effect of SMC in rigorously conducted academic research studies, evidence on the protective effect of SMC in programmatic contexts is more limited. The aim of the analyses presented in our paper was to assess the programmatic impact of SMC in Guinea's context of scaled-up malaria intervention programming, specifically by comparing malaria-related outcomes in districts that had or had not been targeted for SMC between 2015 and 2021. Additionally, to do so, we wanted to use a methodological (i.e., using data routinely collected by Guinea's health management information system [HMIS]) and an analytical approach (i.e., using the compound annual growth rate) that is simple and would allow the National Malaria Control Program (NMCP) to readily monitor SMC effectiveness against standard malariometric indicators as it continues to expand the intervention across the country.

This work directly responds to questions around effectiveness and impact of SMC in Guinea.

2. How were local researchers involved in study design?

The research question and analyses reported in our paper were defined by Guinean co-authors, who are staff of RTI International (MSK, TD, HB, AF), Guinea's Ministry of Health's and NMCP (AC, TG), and the U.S. Agency for International Development (USAID) (LB). Local collaborating researchers and other co-authors were also intricately involved in SMC programmatic efforts between 2015 and 2021.

3. How has funding been used to support the local research team?

USAID | Guinea StopPalu+ funds directly supported MSK, TD, HB and AF and other staff in Guinea. The NMCP and USAID co-authors are funded through their regular employment, and do not require funding through this study.

4. How are research staff who conducted data collection acknowledged?

There was no primary data collection conducted for this study. Health workers in Guinea who implemented SMC programmatic efforts and reported the HMIS data used, as well as communities targeted by SMC are included in the acknowledgements.

5. Do all members of the research partnership have access to study data?

All members of the partnership have access to the raw and cleaned study datasets as well as the final analytical scripts used in the analyses.

6. How was data used to develop analytical skills within the partnership?

DB and RR provided guidance to StopPalu+ staff on the analysis; regular meetings were held with MSK, JLT, and AF during the data analysis phase and initial production of results. These meetings were used to interpret preliminary results and make analytical adjustments accordingly. All authors were involved in the analytical interpretation of the data.

Wider application of the methodology is anticipated to take place in 2024–2025.

7. How have research partners collaborated in interpreting study data?

DB, MSK, C, DT, LB, EM, LF, JLT, AF and RR were involved in interpretation of the results of the data analyses through discussions and presentations. All co-authors reviewed and commented on preliminary results and draft manuscript versions.

8. How were research partners supported to develop writing skills?

Research partners were engaged in the drafting of the manuscript, including its structure, content, and submission to BMJ Global Health. Additional, more broader scientific stature workshops are planned for 2024–2025.

9. How will research products be shared to address local needs?

This paper will be published in an open-access journal, making it accessible to local, regional, and global researchers. It also has been available on a pre-print server to facilitate early sharing of the results for decision-makers. Parts of the analyses were presented in an oral presentation at the 2020 Annual Meeting of the American Society of Tropical Medicine and Hygiene (ASTMH) conference.

The results of this work have already been shared with the Guinean NMCP to inform their future strategic planning.

10. How is the leadership, contribution, and ownership of this work by LMIC researchers recognized within the authorship?

Of the 13 co-authors, 7 are Guinean. MSK, AC and AF were key to the implementation of the SMC programmatic efforts, the design of this study, and contributed to the analysis and interpretation of results. This has been recognized by placing them as second, third authors, and second to last (senior) co-authors; AC is the current NMCP Program Manager and AF was the RTI StopPalu+ Project Director.

11. How have early career researchers across the partnership been included within the authorship team?

There were no early career researchers involved in this study.

12. How has gender balance been addressed within the authorship?

Of the 13 co-authors, three are female (EM, LF, AF) and ten are male (DB, MSK, AC, TG, TD, HB, AP, LB, JLT, RR).

13. How has the project contributed to training of LMIC researchers?

The authorship team is primarily composed of senior public health practitioners and researchers, more than half were either Guinean (MSK, AC, TG, TD, HB, LB, AF) or Guinea-based (EM, JLT).

MSK, TD, HB are mid-career public health practitioners from Guinea who led SMC programmatic efforts and were involved in the design, analysis, and interpretation of the analyses presented here.

14. How has the project contributed to improvements in local infrastructure?

This study has not directly contributed to improvements in local infrastructure.

15. What safeguarding procedures were used to protect local study participants and researchers?

There was no primary data collection as part of this study, therefore this question is not applicable.
